# Characterization of global microRNA expression reveals oncogenic potential of miR-145 in metastatic colorectal cancer

**DOI:** 10.1186/1471-2407-9-374

**Published:** 2009-10-20

**Authors:** Greg M Arndt, Lesley Dossey, Lara M Cullen, Angela Lai, Riki Druker, Michael Eisbacher, Chunyan Zhang, Nham Tran, Hongtao Fan, Kathy Retzlaff, Anton Bittner, Mitch Raponi

**Affiliations:** 1 Johnson and Johnson Research Pty Ltd, Eveleigh, NSW, Australia; 2 Veridex L.L.C., a Johnson and Johnson company, San Diego, CA, USA; 3 Centocor Research & Development, Inc., Radnor, PA, USA; 4 Johnson & Johnson Pharmaceutical Research & Development L.L.C., San Diego, CA, USA; 5 Children's Cancer Institute Australia for Medical Research, PO Box 81 (High Street), Randwick, Sydney, NSW 2031, Australia

## Abstract

**Background:**

MicroRNAs (MiRNAs) are short non-coding RNAs that control protein expression through various mechanisms. Their altered expression has been shown to be associated with various cancers. The aim of this study was to profile miRNA expression in colorectal cancer (CRC) and to analyze the function of specific miRNAs in CRC cells. MirVana miRNA Bioarrays were used to determine the miRNA expression profile in eight CRC cell line models, 45 human CRC samples of different stages, and four matched normal colon tissue samples. SW620 CRC cells were stably transduced with miR-143 or miR-145 expression vectors and analyzed in vitro for cell proliferation, cell differentiation and anchorage-independent growth. Signalling pathways associated with differentially expressed miRNAs were identified using a gene set enrichment analysis.

**Results:**

The expression analysis of clinical CRC samples identified 37 miRNAs that were differentially expressed between CRC and normal tissue. Furthermore, several of these miRNAs were associated with CRC tumor progression including loss of miR-133a and gain of miR-224. We identified 11 common miRNAs that were differentially expressed between normal colon and CRC in both the cell line models and clinical samples. In vitro functional studies indicated that miR-143 and miR-145 appear to function in opposing manners to either inhibit or augment cell proliferation in a metastatic CRC model. The pathways targeted by miR-143 and miR-145 showed no significant overlap. Furthermore, gene expression analysis of metastatic versus non-metastatic isogenic cell lines indicated that miR-145 targets involved in cell cycle and neuregulin pathways were significantly down-regulated in the metastatic context.

**Conclusion:**

MiRNAs showing altered expression at different stages of CRC could be targets for CRC therapies and be further developed as potential diagnostic and prognostic analytes. The identified biological processes and signalling pathways collectively targeted by co-expressed miRNAs in CRC provide a basis for understanding the functional role of miRNAs in cancer.

## Background

Colorectal cancer (CRC) is one of the most frequent cancers and a common cause of cancer-related deaths in the developed world [1]. The overall incidence of CRC is 5% in the general population and the 5-year survival rate ranges from 40% to 60% [2]. Prognosis largely relies upon descriptive staging systems using morphology and histopathology of the tumor [3]. However, even morphologically similar tumors can differ in their underlying molecular changes and tumorigenic potential. The development of CRC from normal epithelial cells to malignant carcinomas involves a multi-step process with accumulation of both genetic and epigenetic changes, leading to a temporal activation of oncogenes and inactivation of tumor suppressor genes that confer a selective advantage to cells containing these alterations [4]. Although most sporadic CRC cases include changes in the WNT, KRAS, TGFβ, β-catenin and p53 pathways, there appears to be added complexity with additional signaling pathways showing accumulated mutations [5,6]. This suggests that alternative factors contribute to CRC and that underlying levels of regulation exist to control the complex cross-talk between different signal transduction pathways.

A number of CRC expression profiling studies on protein coding genes have been performed to better resolve the underlying molecular pathways and to further dissect the different stages of CRC [7,8]. More recently, a newly discovered class of short 22 nucleotide (nt) non-coding RNAs, called microRNAs (miRNAs), have been identified and implicated in cancer initiation and progression [9]. The biogenesis of these small RNAs involves transcription by RNA polymerase II and processing of the primary transcript by the endonuclease Drosha to produce 60-70-nt precursor miRNAs (pre-miRNAs) with imperfect hairpin structures [10,11]. The pre-miRNA is transported into the cytoplasm through exportin 5 [12,13] where it undergoes processing by the RNAse III enzyme Dicer to produce mature miRNAs that are then incorporated into a multiprotein complex [14,15]. These miRNA-containing complexes have been shown to bind to the 3' untranslated region of multiple mRNAs through complementarity between the resident miRNA strand and the target sequence and, based on the degree of homology, direct either translational inhibition or mRNA degradation [16]. To date, there have been 678 human miRNAs identified (miRBase Sequence Database - Release 11) and, through computational models, it has been suggested that there may be greater than 1000 miRNA genes, comprising approximately 3% of the currently known genes in the human genome [17,18]. Moreover, bioinformatic analyses estimate that miRNAs may regulate as many as 30% of the human protein coding genes, suggesting that these small RNAs may act to coordinate the interplay between complex signal transduction pathways [19].

Many miRNAs have been identified as differentially expressed between normal and tumor tissues or cancer cell lines [20]. In CRC, there have been several studies examining the expression patterns of miRNAs [21-27]. The first study showing de-regulation of miRNAs reported the down-regulation of miR-143 and miR-145 as early as the pre-adenomatous polyp stage, suggesting a possible role for these miRNAs in early stages of CRC [24]. Subsequently, a group of 13 miRNAs showing differential expression in CRC tumors was identified with the expression level of miR-31 being correlated with CRC tumor stage [22]. Furthermore, a small number of clinical samples have been screened using a serial analysis of gene expression approach to identify novel miRNAs [23]. Notwithstanding these earlier studies in CRC, there still remains the need to confirm earlier observations in a large set of clinical CRC samples and examine the functional consequences of altered miRNA expression in this cancer.

In this study, we profiled the expression of miRNAs in 45 clinical CRC samples, four matched normal colorectal tissues and eight cell line models. We identified 11 miRNAs commonly changed in expression levels between normal colon and both clinical samples and cell lines. We demonstrate that several miRNAs including miR-1 and miR-31 have evolving expression patterns between stages II and III of CRC and may provide potential prognostic or diagnostic markers for this cancer. Through functional studies, we also show that re-expression of miR-143 or miR-145 leads to tumor suppressor and oncogenic phenotypes in a metastatic CRC model, respectively. The observed oncogenic effects of miR-145 were associated with the down-regulation of the G1/S cell cycle checkpoint and neuregulin pathways in the CRC metastatic setting compared to its isogenically matched non-metastatic model. This pathway analysis may explain the observed oncogenic effects of miR-145 in metastatic CRC compared to its reported tumor suppressor effects in the non-metastatic context.

## Methods

### Cell lines

SW620, SW480, HCT116 and HT29 cell lines were obtained from the ATCC. KM20L2 and KM12C were provided by the NCI-Frederick Cancer DCT Tumor Repository, while cell lines KM20 and KM12SM were supplied by Dr Isaiah J. Fidler (The University of Texas MD Anderson Cancer Center). SW620 and SW480 cells were grown in Dulbecco's Modified Eagle Medium (D-MEM) (Gibco). HCT116 cells were grown in McCoys 5A Media (Gibco) and KM20, KM20L2, KM12C, KM12SM and HT29 cells were grown in RPMI Media 1640 (Gibco). All media was supplemented with 10% fetal bovine serum (JRH Biosciences), 2 mM L-glutamine (Gibco) and Penicillin-Streptomycin solution (0.1 U/mL penicillin and 0.1 μg/mL streptomycin) (Gibco), except for the HT29 cells which were cultured in 0.72 mM L-glutamine.

### Clinical Samples

In total, 49 fresh-frozen human tissue samples were obtained from Genomics Collaborative Inc. (Cambridge MA) or Clinomics Bioscience, Inc (Pittsfield, MA), including 4 normal colon, 4 Stage I, 19 Stage II, 20 Stage III and 2 Stage IV samples (Additional File 1). Fifteen of these samples were profiled in replicate on the Ambion array. In addition, we profiled four matched formalin fixed paraffin embedded (FFPE) samples (B8TMKAN5, NLKEIAE3, S6TRKAX6, and ZIPBBA2E). The median tumor content of all CRC samples was 70%, with no significant difference in tumor content between early stage (I and II) versus late stage (III and IV) disease.

### miRNA profiling

The mirVana Bioarray (Ambion, version 1) that contains 287 human miRNA probes was employed to identify colorectal cancer miRNA signatures [28]. MiRNA was isolated from 5 ug of total RNA from colorectal samples using the mirVana isolation kit (Ambion) for snap-frozen samples and the RecoverAll™ Total Nucleic Acid Isolation Kit for FFPE samples (Ambion). All samples were then fractionated by polyacrylamide gel electrophoresis (Flash-Page Ambion) and small RNAs (< 40 nt) were recovered by ethanol precipitation with linear acrylamide. Quantitative RT-PCR (QPCR) of miR-16 was used to confirm miRNA enrichment prior to miRNA array analysis.

The small RNAs from all samples were subject to poly(A) polymerase reaction wherein amine modified uridines were incorporated (Ambion). The tailed samples were then fluorescently labeled using the amine-reactive Cy3 or Cy5 (Invitrogen). One- or two-color hybridizations were performed for the clinical CRC or cell line profiling experiments, respectively. For 2-color experiments, cell line miRNA was directly compared to normal colon RNA (Ambion). The fluorescently labeled RNAs were purified using a glass-fiber filter and eluted (Ambion). Each sample was then hybridized to the Bioarray slides for 14 hours at 42°C (Ambion). The arrays were then washed and scanned using an Agilent 2505B confocol laser microarray scanner (Agilent) and data was obtained using the Expression Analysis software (Codelink, version 4.2). The mirVana microarray data have been deposited in the NCBI GEO and are accessible through GEO series accession no. GSE10259.

### Statistical Analysis

The data were analyzed using the R software package. The miRNA expression data were quantile normalized prior to determining differential gene expression. Replicate samples and probe values were averaged and the Student t-test was performed to find genes that vary significantly across sample groups. Genes were selected if the median normalized signal intensity was greater than 100 (75^th ^percentile of median signal) for at least one group, with a mean change > 1.5-fold and a p-value < 0.05. A one-way ANOVA was used to evaluate miRNA expression level between normal and different cancer stages. Both probe level and gene level data analysis was performed for all group comparisons.

To compare miRNA profiles measured between matched FFPE and fresh frozen tissue, or QPCR versus mirVana array data, Taqman Ct values greater than 35 (approaching background expression levels) were first removed and then linear regression and Pearson correlation calculations were performed.

### miRNA QPCR

QPCR was performed using the ABI early access miRNA Taqman panel to verify miRNA expression profiles [29]. This included 169 individual assays as listed in Additional File 2. Ten ng of total RNA was converted to cDNA using the High Capacity DNA Archive kit and 3 ul of 5× RT primer according to the manufacturer's instructions (Ambion). The 15 μl reactions were incubated in a thermocycler for 30 min at 16°C, 30 min at 42°C, 5 min at 85°C and held at 4°C. All reverse transcriptase reactions included no template controls. QPCR was performed using a standard Taqman^® ^PCR kit protocol on an Applied Biosystems 7900 HT Sequence Detection System. The 10 μl PCR reaction included 0.66 μl RT product, 1 μl Taqman miRNA assay primer and probe mix, 5 μl Taqman 2× Universal PCR master mix (No Amperase UNG) and 3.34 μl water. The reactions were incubated in a 384 well plate at 95°C for 10 mins, followed by 40 cycles of 95°C for 15 sec, and 60°C for 2 min. All QPCR reactions included a no cDNA control and all reactions were performed in triplicate.

### Plasmid constructs

The plasmid pSilencer 2.1 (Ambion) was modified within the multiple cloning site to introduce unique restriction sites and a RNA polymerase III transcriptional terminator (TTTTTT). The following oligonucleotides were synthesized (Sigma), annealed and ligated to pSilencer 2.1, pre-digested with BamHI and HindIII, to produced pSilencer 2.1 Term: pSilU6upper 5'GATCCCTCGAGTCTAGATTTTTTGGAAA and pSilU6lower 5'AGCTTTTCCAAAAAATCTAGACTCGAGG. The DNA encoding pre-miR-143 was PCR-amplified from human genomic DNA using primers 143F (5'CGGGATCCCGGAGAGGTGGAGCCCAGGTC) and 143R (5'GCTCTAGACAGCATCACAAGTGGCTGA), digested with BamHI and XbaI and ligated to pSilencer 2.1Term to produce the miR-143 expression plasmid. For miR-145, genomic DNA was recovered as a PCR amplicon using primers 145F (5'CGGGATCCCAGAGCAATAAGCCACATCC) and 145R (5'GCTCTAGACTCTTACCTCCAGGGACAGC), digested with BamHI and XbaI and ligated into pSilencer 4.1 under control of the CMV promoter.

### Generation of stable cell lines

To generate stable clones using pSilencer 2.1 (miR-143) and pSilencer 4.1 (miR-145) plasmids, 1-5 × 10^6 ^SW620 cells were seeded in a single well of a 6-well plate. Upon reaching 80-90% confluence cells were transfected with 4 ug plasmid DNA using Lipofectamine 2000 according to the manufacturer's instructions. Cells were diluted and plated in fresh medium containing 500 ug/ml hygromycin. After 14 days of selection, independent clones were picked, expanded and screened for expression of the specific miRNA(s) encoded by the transfected plasmid.

### Antisense experiments

Biotinylated 2'-O-methyl antisense miR-145 RNA or controls were delivered to SW620 using Lipofectamine 2000 (Invitrogen) as detailed in the Supplementary Methods. Transfection efficiency was at least 80% in all experiments. All experiments were performed in triplicate.

### Cell proliferation assay

SW620 cells (3 × 10^3 ^cells/well) were seeded in 96-well plates in serum-containing media. Cell viability analysis was conducted on day 0 (day of seeding) and on day 5. A total of 20 μL of Cell Titer-Blue reagent (Promega) was added to each well and the plate incubated at 37°C for 2 hours. Fluorescence was measured using the FLUOstar OPTIMA microplate reader (BMG Labtech). To conduct serum-free analysis, the media was changed to serum-free media on day 1.

### Rapid soft agar assay

2× Iscove's Modified Dulbecco's Medium (IMDM) (Gibco) was supplemented with 0.6% Sodium Bicarbonate, 20% fetal bovine serum (JRH Biosciences), 4 mM L-glutamine (Gibco), 2× Non Essential Amino Acid Solution (Gibco), 2% Sodium Pyruvate (Gibco) and Penicillin-Streptomycin solution (0.1 U/mL penicillin and 0.1 μg/mL streptomycin) (Gibco). 2× IMDM was mixed at a ratio of 1:1 with 1.2% Bacto Agar (55°C) and 50 μL was added per well to a 96-well plate to produce a pre-layer for the assay. Ten μL of cell suspension containing SW620 cells (3 × 10^3^), 20 μL of 2× IMDM and 30 μL 0.8% Bacto agar (55°C) was mixed and transferred to the solidified pre-layer in each well. The semi-solid feeder layer was prepared by mixing 25 μL of 2× IMDM and 25 μL of 1.2% Bacto Agar (55°C) and layered on top of the solidified cell layer. The cells were left to grow in a CO_2 _incubator at 37°C for 7 days. Proliferation and cell viability were scored using the Cell Titer-Blue reagent (Promega).

### Live cell imaging

The Nikon Diaphot biological microscope and the Nikon E995 digital camera were used to capture the cell morphology images.

### Antisense-mediated suppression of miR-145

A total of 100 nM of biotinylated 2'-O-methyl antisense miR-145 RNA (5' AAG GGA UUC CUG GGA AAA CUG GAC 3') (IDT) or the reverse control (5' CAG GUC AAA AGG GUC CUU AGG GAA 3') (IDT) were transfected with Lipofectamine 2000 (Invitrogen) into 2 × 10^6 ^SW620/miR-145 cells. At 48 hours post-transfection, the cells were harvested and total RNA extracted using TRIzol (Invitrogen) according to the manufacturer's instructions. 100 ug of total RNA was subjected to three sequential depletions using 250 ug of Dynabeads M-280 Streptavidin coated magnetic beads (Invitrogen). Each depletion involved washing the beads twice with 0.1 M NaOH, 0.05 M NaCl solution and then once with 0.1 M NaCl. The beads were resuspended in 0.1 M NaCl and 250 ug beads were added to the RNA sample. The sample was agitated for 20 minutes at 4°C. The beads were separated from the RNA solution using a magnetic separation apparatus (Promega) and the solution (containing the depleted RNA) was removed from the beads. The depleted RNA was ethanol precipitated and analyzed for miR-145 and U6 snRNA expression by Northern analysis.

Transfection efficiency of the FAM-labelled 2'O-methyl antisense miR-145 RNA oligonucleotide was conducted in parallel with the depletion experiments. A total of 100 nM of FAM-labelled 2'O-methyl antisense miR-145 RNA (IDT) was transfected with Lipofectamine 2000 (Invitrogen) into 1.2 × 10^5 ^SW620 cells expressing miR-145. At 48 hours post-transfection, the cells were harvested and subjected to FACS analysis.

### Northerns for miRNAs

TRIzol extraction of total RNA was carried out according to the manufacturer's specifications (Invitrogen). Briefly, cells were washed with PBS and 5 mL TRIzol reagent added and cells incubated for 5 minutes at room temperature. After adding 1 mL chloroform, cells were shaken vigorously for 15 seconds by hand. The samples were centrifuged and the aqueous layer transferred to a 15 mL falcon tube containing 2.5 mL isopropanol. The samples were incubated at room temperature for 20 minutes, centrifuged as above to pellet the RNA and resuspended with 1 mL 75% EtOH. RNA was pelleted by centrifugation, air-dried and resuspended in 50 μL DEPC water (Ambion).

Northern analysis was conducted using 15% PAGE-Urea gels, prepared using the SequaGel Sequencing System (National Diagnostics), and electrophoresis was carried out using the MiniProtean II gel electrophoresis apparatus (BioRad). A total of 40 μg RNA was added to 10 μl RNA loading dye (2× solution of 95% Formamide, 18 mM EDTA, and 0.025% SDS, Xylene Cyanol, and Bromophenol Blue) and incubated at 65°C for 10 minutes. The samples were loaded onto the 15% PAGE-Urea/TBE gel and electrophoresed in 1× TBE at 100 V until the bromophenol blue dye reached the bottom of the gel. The RNA was transferred onto the Hybond-N+ membrane (GE Healthcare) using the Mini Trans-Blot Electrophoretic Transfer Cell (BioRad) in 0.5× TBE buffer with 80 V for 1 hour. The RNA was cross-linked to the membrane using the UV Stratalinker 1800 (1200 joules) (Stratagene).

Membranes were pre-hybridized in 10 mL Express hybridization solution (Clontech) at 37°C. The Starfire oligonucleotide probe was boiled for 1 minute and then added to the hybridization solution. After overnight hybridization at 37°C, the hybridization solution was removed and the membrane rinsed three times with 2× SSC/0.1% SDS and further washed with 2× SSC/0.1% SDS solution at 37°C for 15 minutes. The membrane was exposed to a Storage Phosphor Screen (GE Healthcare) overnight and imaged using the Typhoon Trio machine (GE Healthcare). The membrane was stripped of the bound probe by pouring boiling 0.1% SDS directly onto the membrane and then allowing the solution to slowly cool over a 30 minute period.

Custom Starfire oligonucleotide probes were synthesized by Integrated DNA Technologies (IDT). The lyophilized oligonucleotide probes were diluted to 100 μM stock solution in 1× TE pH 8.0. The labelling reaction included 1× exo^- ^reaction buffer (NEB), 1 μL Starfire Universal template oligonucleotide (IDT) and 0.5 pmol Starfire oligonucleotide probe. The reaction mix was boiled for 1 minute and then allowed to cool to room temperature for 5 minutes before adding 50 μCi α-^32^P-dATP (10 mCi/mL, 6000 Ci/mmol) (Perkin-Elmer) and 5 U exo^- ^Klenow DNA polymerase (NEB) and incubating at room temperature for 90 minutes. The reaction was stopped by the addition of 40 μL 10 mM EDTA. The unincorporated α-^32^P-dATP was removed from the reaction mix using MicroSpin G-25 columns (GE Healthcare) according to manufacturer's instructions. Prior to use, the probe was boiled for 1 minute. Sequences of Starfire probes used are shown in Additional File 3.

The U6 snRNA oligonucleotide probe (5' AAC GCT TCA CGA ATT TGC GT 3') was end labeled using 20 pmole oligonucleotide probe, 1× T4 polynucleotide buffer (NEB), 50 μCi γ-^32^P-dATP (10 mCi/mL, 6000 Ci/mmol) (Perkin Elmer) and 10 U T4 polynucleotide kinase (NEB), in a final volume of 20 μL. The probe was incubated for 30 minutes at 37°C. The reaction was stopped by the addition of 40 μL 10 mM EDTA. The unincorporated γ-^32^P-dATP was removed from the reaction mix using MicroSpin G-25 columns (GE Healthcare) according to manufacturer's instructions. Prior to use, the probe was boiled for 5 minutes.

### MiRNA target prediction and functional analysis

META-miR:Target Inference (MAMI - Meta prediction of microRNA targets, http://mami.med.harvard.edu/) was used for miRNA target prediction. This tool performs a meta-analysis of mRNA targets derived from all available target prediction algorithms. Initially, a predicted target gene set was generated by MAMI from differentially expressed miRNAs. Sensitivity and specificity thresholds were set in order to identify approximately 300 or more target mRNAs. Second, gene ontology enrichment analysis of biological processes (hierarchical level 5) was applied on this set of predicted miRNA target genes, in which the human genome was used as a reference set [30]. Gene Ontology terms or KEGG pathway enrichment was determined by Fisher Exact test with a p value less than 0.05. In addition, enrichment analysis of signaling pathways, comparative analysis of biological functions and disease categories were performed to further investigate functional characterization of the enriched target genes using Ingenuity Pathway Analysis (Ingenuity Systems).

## Results

### Profiling miRNA in clinical CRC samples

**Figure 1:**
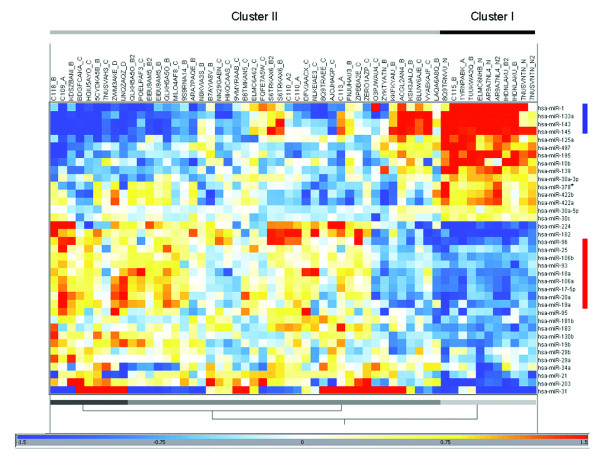
**Two-way hierarchical clustering of CRC and normal colorectal tissue using 37 differentially expressed miRNAs**. The hypergeometric mean of the log2 signal intensity was calculated across all samples using a euclidean distance metric with complete linkage. Red and blue indicates miRNAs expressed at a high or low level relative to other samples in the dataset, respectively. The miR-143-145 and miR-17-92 clusters are indicated by vertical blue and red bars, respectively. Samples are grouped into two main clusters: Cluster I primarily represents normal colorectal tissue, and clusters II represents CRC samples. Replicate samples are indicated by the suffix "2".

We initially profiled 64 samples from 49 patients with colorectal cancer (4 matched adjacent normal colorectal tissues, 4 Stage I, 19 Stage II, 20 Stage III, and 2 Stage IV CRC; plus 15 replicates) for differential miRNA expression between normal and tumor tissues and also between stage II and stage III disease (Additional File 1). A total of 37 differentially expressed miRNAs were identified between clinical CRC tissues and adjacent normal colorectal tissue (Table 1). Most of these miRNAs are associated with chromosomal regions that are known to have frequent gains or losses in CRC [31]. Supervised hierarchical clustering of the 37 miRNAs showed that many were co-ordinately expressed, including the miR-143-145 and miR-17-92 clusters, which were consistently down- or up-regulated in CRC, respectively (Fig. 1).

**Table 1:** miRNAs differentially expressed between CRC and normal colorectal tissue.

miRNA	Normal^a^	CRC^a^	p-value	fold change^b^	Location^c^	CRC Fragile site^d^
hsa-miR-20a	9.2	10.3	2.0E-03	2.1	13q31.3	gain
hsa-miR-18a	7.6	8.6	2.9E-03	2.0	13q31.3	gain
hsa-miR-19a	7.7	8.7	2.3E-03	1.9	13q31.3	gain
hsa-miR-17-5p	10.5	11.4	1.5E-03	1.9	13q31.3	gain
hsa-miR-19b	11.0	11.8	3.4E-03	1.8	13q31.3	gain
hsa-miR-203	8.4	9.7	2.2E-02	2.6	14q32.33	loss
hsa-miR-21	13.0	14.5	6.0E-06	2.9	17q23.2	gain
hsa-miR-34a	9.5	10.3	1.5E-02	1.7	1p36.22	loss
hsa-miR-181b	8.5	9.2	2.2E-04	1.7	1q31.1 or 9q33.3	gain (1q)
hsa-miR-29b	9.7	10.5	4.9E-03	1.8	1q32.2 or 7q32.3	gain
hsa-miR-130b	7.1	7.8	1.2E-03	1.7	22q11.21	loss
hsa-miR-95	6.1	6.8	1.1E-02	1.6	4p16.1	loss
hsa-miR-106b	9.5	10.3	1.0E-04	1.7	7q22.1	gain
hsa-miR-93	9.9	10.5	3.4E-03	1.6	7q22.1	gain
hsa-miR-25	9.0	9.6	1.4E-02	1.6	7q22.1	gain
hsa-miR-182	7.2	8.7	2.8E-03	2.8	7q32.2	gain
hsa-miR-96	6.7	7.7	8.4E-03	2.0	7q32.2	gain
hsa-miR-183	5.9	6.8	2.3E-03	1.8	7q32.2	gain
hsa-miR-29a	12.1	12.9	3.6E-04	1.7	7q32.3	gain
hsa-miR-31	6.4	8.7	2.6E-03	5.0	9p21.3	
hsa-miR-106a	10.7	11.7	5.1E-04	2.0	Xq26.2	
hsa-miR-224	6.9	8.4	1.1E-03	2.8	Xq28	
hsa-miR-30a-5p	11.6	11.0	1.6E-05	0.7	6q13	loss
hsa-miR-30a-3p	7.1	6.3	7.3E-04	0.5	6q13	loss
hsa-miR-378*	7.6	6.5	1.3E-05	0.4	5q32	loss
hsa-miR-422b	10.9	9.6	8.3E-05	0.4	5q32	loss
hsa-miR-143	14.1	12.6	1.0E-02	0.4	5q32	loss
hsa-miR-145	15.0	13.2	1.2E-03	0.3	5q32	loss
hsa-miR-10b	11.0	10.1	2.1E-02	0.5	2q31.1	
hsa-miR-30c	10.9	10.3	4.0E-04	0.7	1p34.2 or 6q13	loss
hsa-miR-125a	11.2	10.2	6.4E-03	0.5	19q13.41	gain
hsa-miR-1	9.0	7.0	1.6E-03	0.2	18q12.3 or 20q13.33	loss (18q) or gain (20q)
hsa-miR-133a	10.0	7.6	6.5E-04	0.2	18q12.3 or 20q13.33	loss (18q) or gain (20q)
hsa-miR-497	9.3	8.1	9.8E-04	0.4	17p13.1	loss
hsa-miR-195	11.1	9.5	4.2E-05	0.3	17p13.1	loss
hsa-miR-422a	10.0	8.8	7.3E-05	0.4	15q22.31	loss
hsa-miR-139	7.6	6.2	1.1E-05	0.4	11q13.4	loss

a. normalized median signal intensity (log2)

b. cancer:normal

c. Chromosomal location of some miRNAs equivocal since MirVana Bioarray does not discriminate between pre-miRNA precursors.

d. Frequent chromosomal gains or lossesd in CRC as summarized from [31].

**Figure 2:**
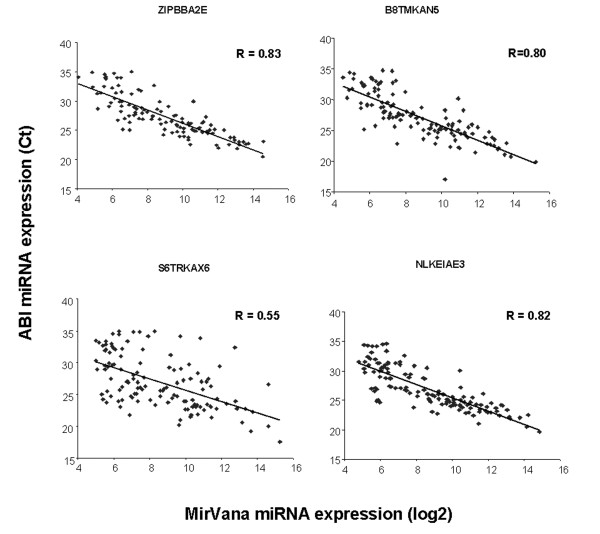
**Correlation of miRNA expression comparing the mirVana Bioarray and ABI Taqman platforms**. Linear regression was performed on miRNAs that were measured on both platforms. Four different CRC clinical samples were examined. Taqman and Ambion miRNA expression is measured in Ct and log_2 _units, respectively. The Pearson correlation was calculated for each comparison.

**Figure 3:**
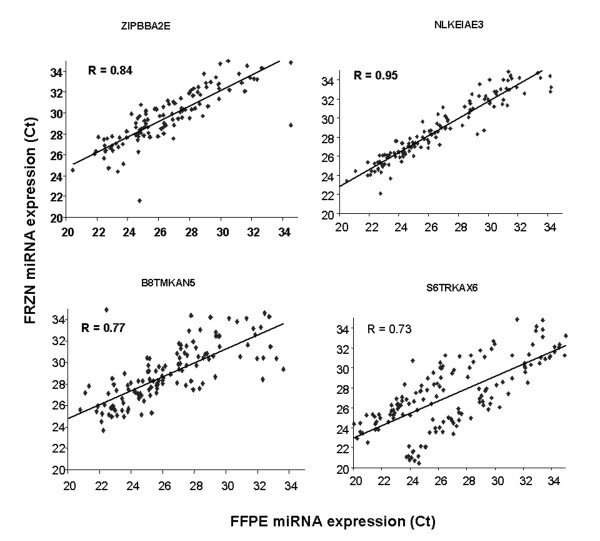
**miRNA expression in fresh frozen versus formalin-fixed paraffin embedded CRC samples**. Taqman miRNA expression assays were performed on 169 miRNAs from 4 matched fresh frozen and FFPE samples and Ct measurements were compared by linear regression. The Pearson correlation was calculated for each comparison.

To validate the MirVana miRNA expression profiles on a different platform we performed Taqman QPCR analysis on 169 miRNAs in four of the clinical CRC samples (Fig. 2). The median correlation of miRNA expression between platforms was 0.81. Hierarchical clustering also demonstrated that replicate samples were proximal to each other, further indicating highly reproducible results. In addition, miRNAs could be reproducibly detected in four matched FFPE and fresh frozen CRC samples using the ABI QPCR platform with a median correlation of 0.81 (Fig. 3). Reproducible detection of miRNAs in FFPE samples was an important observation since these samples are widely available for diagnostic testing.

In addition, we investigated whether any miRNAs were associated with CRC progression. A set of 22 miRNAs were found to be differentially expressed between normal and early stage (mostly stage II) CRC including increases in miR-21 and miR-224 and decreases in miR-133a and miR-145 (Table 2). Six miRNAs showed significant differential expression between early and late stage (mostly stage III) disease including an increase in miR-31 expression (Table 3). Both miR-133a and miR-378* were significantly reduced from normal tissue through stages II and III of CRC. This indicates that loss of these miRNAs is associated with later stage disease progression.

**Table 2:** miRNAs differentially expressed between normal colon and early stage colorectal cancer (Stages I and II).

miRNA	Normal	Stage I/II	Fold change	p-value
hsa-miR-224	6.89	8.67	3.45	1.80E-02
hsa-miR-21	13.02	14.49	2.78	1.52E-04
hsa-miR-34a	9.48	10.43	1.92	1.92E-02
hsa-miR-106a	10.68	11.57	1.85	2.88E-02
hsa-miR-18a	7.51	8.38	1.83	3.32E-02
hsa-miR-29b	9.70	10.56	1.82	3.27E-03
hsa-miR-19b	10.84	11.69	1.80	2.77E-02
hsa-miR-106b	9.45	10.26	1.76	1.37E-02
hsa-miR-20a	9.28	10.06	1.72	3.75E-02
hsa-miR-29a	12.14	12.89	1.68	9.84E-04
hsa-miR-181b	8.41	9.11	1.63	9.47E-03
hsa-miR-19a	7.78	8.45	1.60	2.58E-02
hsa-miR-95	6.12	6.79	1.59	3.33E-02
hsa-miR-516-3p	4.87	5.52	1.57	3.20E-02
hsa-miR-378*	7.53	6.85	0.62	1.87E-03
hsa-miR-422b	10.88	10.02	0.55	4.91E-03
hsa-miR-422a	10.05	9.19	0.55	9.57E-03
hsa-miR-30a-3p	7.14	6.24	0.54	1.51E-02
hsa-miR-139	7.62	6.17	0.37	5.75E-03
hsa-miR-195	11.04	9.52	0.35	2.37E-02
hsa-miR-145	15.11	13.46	0.32	4.30E-02
hsa-miR-133a	10.51	7.64	0.14	3.00E-02

Fold change = cancer:normal

**Table 3:** miRNAs differentially expressed in early (I and II) vs late stage (III and IV) disease.

Sample ID	Stage I/II	Stage III/IV	Fold change	p-value
hsa-miR-31	7.11	9.97	7.22	1.53E-03
hsa-miR-7	7.77	8.85	2.11	1.96E-02
hsa-miR-99b	8.74	8.12	0.65	3.64E-03
hsa-miR-378*	6.85	6.23	0.65	3.02E-02
hsa-miR-133a	7.64	6.96	0.63	1.64E-02
hsa-miR-125a	10.64	9.76	0.54	2.69E-03

Fold change = Stage III/IV:Stage I/II

### Profiling miRNA in CRC cell lines

**Figure 4:**
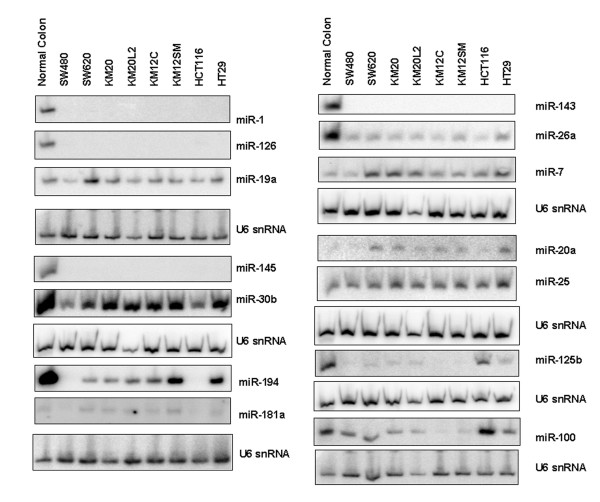
**Expression of 14 miRNAs in 8 CRC cell lines and normal colon total RNA**. Northern blots were performed using U6 snRNA as a normalization control.

To identify a CRC cell line for functional analysis of differentially expressed miRNAs, and to examine the relationship between miRNA expression in clinical samples and cell lines, we compared miRNA expression between normal colonic epithelial cells and four CRC cell line models (SW480, SW620, KM12C, KM12SM). From this analysis, 43 miRNAs were identified as either 2-fold up- or 2-fold down-regulated in at least one of the four CRC cell lines (Table 4). To validate the cell line microarray data, we performed Northern blot analyses on 14 of the 43 miRNAs in these and an additional 4 cell lines (Fig. 4). Of those miRNAs that were identified in the clinical samples, only 11 were in common with the 43 deregulated in the cell line models (Table 5). This indicated that the findings from clinical samples are not completely reproduced in cell line models. However, the commonly deregulated miRNAs included miR-31, members of the miR-17-92 cluster, miR-1, miR-143 and miR145, thereby validating the findings of previous studies.

**Table 4:** MiRNAs differentially expressed in four CRC cell lines vs normal colorectal tissue.

MiRNA	SW480	SW620	KM12C	KM12SM	Expression
hsa-let-7e	1.06	0.57	0.68	0.48	down
hsa-let-7f-1	0.46	0.41	0.40	0.36	down
hsa-let-7f-2	0.41	0.41	0.37	0.34	down
**hsa-miR-1**	0.33	0.51	0.56	0.29	**down**
**hsa-miR-100**	0.56	0.92	0.64	0.41	**down**
hsa-miR-106a	0.92	3.78	2.48	2.14	up
hsa-miR-107	0.48	1.00	1.04	1.03	down
hsa-miR-10a	2.38	0.98	0.31	0.50	up/down
hsa-miR-122a	0.82	0.57	0.59	0.43	down
hsa-miR-125a	0.53	0.43	0.26	0.30	down
**hsa-miR-125b**	0.12	0.37	0.07	0.16	**down**
**hsa-miR-126**	0.17	0.09	0.06	0.07	**down**
hsa-miR-140	2.27	1.06	1.58	1.43	up
hsa-miR-141	0.30	0.18	1.64	1.55	up/down
**hsa-miR-143**	0.03	0.01	0.02	0.01	**down**
**hsa-miR-145**	0.05	0.02	0.03	0.02	**down**
hsa-miR-150	0.34	0.32	0.38	0.24	down
hsa-miR-151-3p	0.62	0.45	0.79	0.50	down
hsa-miR-15b	2.14	1.45	1.89	1.46	up
hsa-miR-17-5p	0.76	3.05	2.31	1.80	up
hsa-miR-18a	0.55	2.22	2.16	1.79	up
**hsa-miR-181a**	0.47	1.49	1.27	1.38	**up/down**
hsa-miR-186	0.47	2.11	0.67	0.60	down
hsa-miR-188	0.72	1.27	1.37	0.46	down
hsa-miR-192	0.02	0.08	0.18	0.28	down
**hsa-miR-194**	0.03	0.10	0.27	0.38	**down**
**hsa-miR-19a**	0.59	2.48	1.79	1.25	**up**
**hsa-miR-20a**	0.75	3.29	1.99	1.99	**up**
hsa-miR-200a	0.26	0.97	1.59	1.93	up/down
hsa-miR-200b	0.44	0.97	1.64	2.20	up/down
hsa-miR-215	0.02	0.02	0.05	0.06	down
hsa-miR-22	0.46	0.15	0.28	0.21	down
**hsa-miR-25**	2.23	1.92	1.55	1.52	**up**
**hsa-miR-26a**	0.47	0.25	0.18	0.26	**down**
hsa-miR-298	2.38	1.00	1.69	1.41	up
hsa-miR-30a-3p	0.50	0.46	0.64	0.73	down
**hsa-miR-30b**	0.34	0.42	0.83	1.02	**down**
hsa-miR-31	0.31	0.20	1.92	2.77	up/down
hsa-miR-320	1.69	1.47	2.46	2.28	up
**hsa-miR-7**	1.58	2.77	2.45	1.83	**up**
hsa-miR-92	1.75	4.23	2.41	2.25	up
hsa-miR-93	2.16	2.27	2.31	2.45	up
hsa-miR-99a	1.23	0.69	0.70	0.49	down

miRNAs in bold were validated by Northern blot analysis

**Table 5:** MiRNAs commonly deregulated in CRC clinical samples and CRC cell lines.

miRNA	fold change	location	CRC Fragile site*
hsa-miR-20a	up	13q31.3	gain
hsa-miR-19a	up	13q31.3	gain
hsa-miR-17-5p	up	13q31.3	gain
hsa-miR-93	up	7q22.1	gain
hsa-miR-25	up	7q22.1	gain
hsa-miR-31	up	9p21.3	
hsa-miR-106a	up	Xq26.2	
hsa-miR-143	down	5q32	loss
hsa-miR-145	down	5q32	loss
hsa-miR-125a	down	19q13.41	gain
hsa-miR-1	down	18q12.3 or 20q13.33	loss (18q) or gain (20q)

* Summarized from [41-43]

### miR-143 has a tumor suppressor effect in metastatic CRC cells

**Figure 5:**
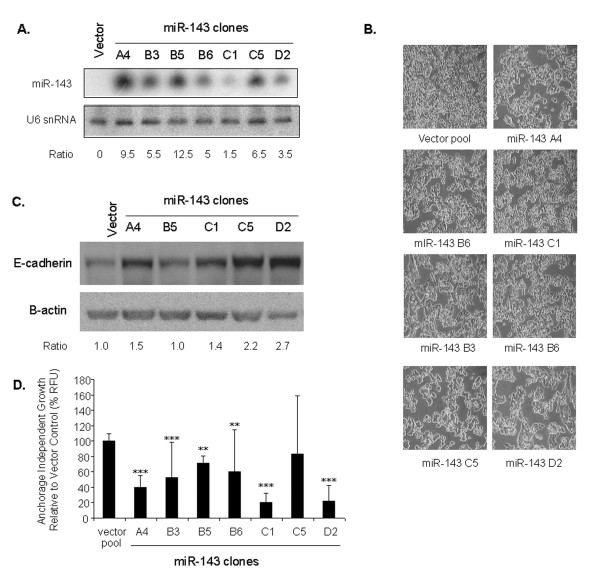
**Over-expression of miR-143 in the SW620 cell line affects cell morphology and proliferation**. (A) MiR-143 genomic DNA was cloned under control of the U6 promoter (in pSilencer 2.1) and transfected into SW620 cells. Seven stable SW620 clones were identified that expressed miR-143 (A4, B3, B5, B6, C1, C5, and D2). U6 snRNA was used as a loading control. (B) The seven SW620/miR-143 clones were examined for cell morphology compared to vector control. (C) Western analysis of the SW620/miR-143 clones and control cells shows the steady-state levels of E-cadherin. E-cadherin ratios were calculated using β-actin as a normalization control. (D) Seven SW620/miR-143 clones were assayed for proliferation/metabolic activity compared with vector control when grown in the absence (open bars) or presence of serum (solid bars). (E) The same clones were examined for anchorage-independent cell growth in the rapid soft agar assay in the presence of serum. Two independent experiments were performed. ** p < 0.01, *** p < 0.001.

MiR-143 and miR-145 consistently displayed reduced expression levels in CRC clinical samples and were undetectable in CRC cell line models. Over-expression of miR-143 and miR-145 has previously been shown to result in a tumor suppressor effect in non-metastatic CRC cell lines [21,27,32]. In contrast, in the current study we examined the activity of these miRNAs in the metastatic CRC context using the SW620 cell line model (Fig. 5). Seven stable clones expressing miR-143 were isolated and each displayed a cell clumping phenotype, which was associated with increased E-cadherin protein expression (Fig. 5A-C). No difference in cell proliferation rates was observed (data not shown), however, reduced anchorage-independent growth was demonstrated in all clones (Fig. 5D). There was no correlation between the level of miR-143 expression, E-cadherin, or the degree of growth inhibition.

### miR-145 has an oncogenic effect in metastatic CRC cells

**Figure 6:**
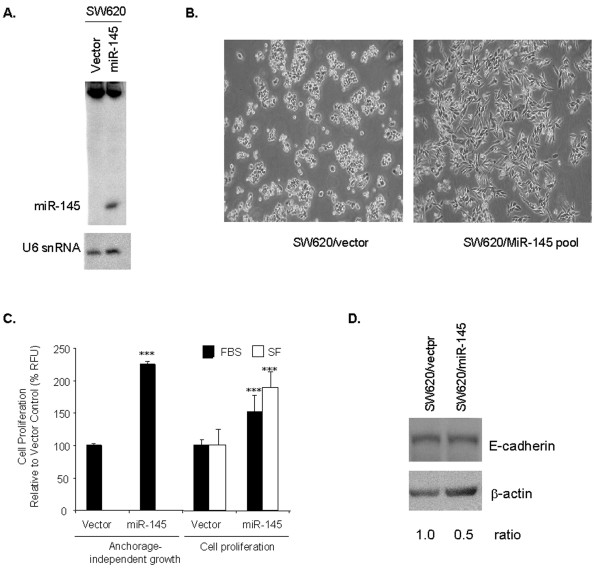
**Over-expression of miR-145 in SW620 cell line affects cell morphology and proliferation**. (A) The genomic region surrounding the miR-145 gene was PCR-amplified and cloned into pSilencer 4.1 under control of the CMV promoter. Mature miR-145 was detected by Northern analysis in a pooled population of SW620 cells following transfection. U6 snRNA was used as a loading control. (B) A major distinguishing feature of the cell population over-expressing miR-145 was the change in cell morphology from the round single cells of SW620 to elongated cells with extended processes typical of fibroblast-like cells. (C) The miR-145-expressing SW620 cell population showed a two-fold increase in anchorage-independent growth when grown in the presence of serum and a greater than 50% increase in cell proliferation/metabolic activity when grown in the presence (solid bars) or absence (open bars) of serum. *** p < 0.001. (D) Western analysis of the SW620/miR-145 cells and control cells shows the steady-state levels of E-cadherin were 50% lower in SW620 cells expressing the mature miR-145. FBS = fetal bovine serum, SF = serum-free.

A stable pooled population of SW620 cells expressing detectable steady-state levels of mature miR-145 (designated SW620/miR-145) was generated (Fig. 6A). The level of miR-145 approximated those observed in normal colonic epithelial tissue as determined by U6 snRNA-normalized Northern analysis (Fig. 4 and Fig. 6a). A major distinguishing feature of the SW620/miR-145 cell population was the change from the round single cells of SW620 to elongated cells with extended processes typical of fibroblast-like cells (Fig. 6B). The SW620/miR-145 cells also showed a 50% to 95% increase in cell proliferation/metabolic activity when grown in the presence or absence of serum, respectively (Fig. 6C). A two-fold increase in anchorage-independent growth when grown in the presence of serum was also observed (Fig. 6C). The epithelial cell marker E-cadherin was also reduced by 50% in SW620/miR-145 cells compared to controls (Fig. 6D), which is consistent with the mesenchymal-like cell morphology and increased proliferation observed for SW620/miR-145 cells.

**Figure 7:**
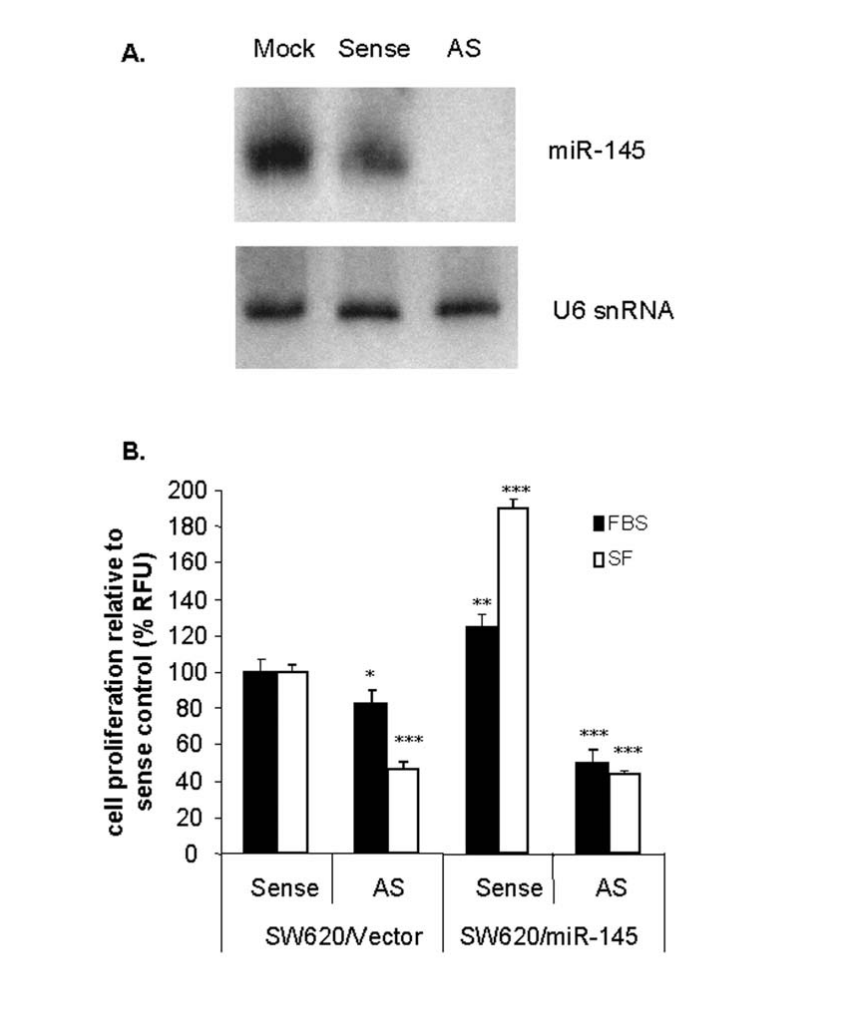
**Antisense-mediated reversion of miR-145-induced proliferation**. (A) Total RNA from treated samples showed that miR-145 was depleted in cells receiving the miR-145-specific 2'Ome antisense RNA but not in the mock treated, or miR-145 sense treated controls. (B) As expected, SW620/miR-145 expressing pools showed increased proliferation compared to vector controls in the presence (solid bars) or absence (open bars) of serum. When treated with miR-145 antisense RNA, a reduction in proliferation was seen in both SW620/vector and SW620/miR-145 pools. Three independent experiments were performed. * p < 0.05; ** p < 0.01, *** p < 0.001.

To confirm that the changes in SW620/miR-145 cells were due to miR-145 expression we performed miR-145-specific 2'O-methyl (2'Ome) antisense RNA knock down experiments. MiR-145 was depleted in SW620/miR-145 cells receiving the miR-145-specific 2'Ome antisense RNA but not in the sense control (Fig. 7A). Over-expression of miR-145 in the presence of sense control RNA resulted in increased cell proliferation in serum, and more markedly in serum-free medium (Fig. 6C and 7B). However, when antisense miR-145 RNA was transfected into SW620/miR-145 cells there was a reversion of the high proliferative potential of the cells (Fig. 7B). These results indicated that it was the specific ectopic expression of miR-145 that induced changes in cell differentiation and increased the proliferative potential of this cell line model.

### Genetic network analysis of miR-143 and miR-145 targets

The above data indicated that over-expression of miR-145 leads to an oncogenic phenotype in metastatic CRC cells, the opposite effect of miR-143. We therefore performed bioinformatic analyses to help elucidate the difference between miR-143 and miR-145 biology. To that end we investigated the biological pathways regulated by these miRNAs by performing gene set enrichment analysis of their predicted mRNA targets [33]. To validate the approach we first examined the predicted 1683 mRNA targets of the 37 miRNAs differentially expressed between normal colon mucosa and CRC tissue. The significant enrichment of CRC associated pathways, including WNT/β-catenin, TGF-β, and ERK/MAPK, indicated that this approach was valid (Table 6). When this analysis was performed specifically for miR-145 and miR-143 targets, no significant overlap of pathways was found indicating that the two co-expressed miRNAs target different biological pathways, possibly explaining the opposing phenotypes observed.

**Table 6:** Pathways targeted by CRC miRNAs

		p-value
	
Pathway	miRNA targets enriched	Known colon cancer genes
B Cell Receptor Signaling	3.02E-08	3.16E-07
IL-6 Signaling	4.27E-07	1.38E-04
Glucocorticoid Receptor Signaling	7.41E-07	6.17E-10
ERK/MAPK Signaling	1.51E-06	2.51E-03
Ephrin Receptor Signaling	4.90E-06	5.62E-04
Ceramide Signaling	1.02E-05	8.32E-07
TGF-beta Signaling	2.63E-05	2.04E-06
Neurotrophin/TRK Signaling	3.02E-05	1.35E-04
Axonal Guidance Signaling	5.01E-05	6.17E-06
SAPK/JNK Signaling	5.37E-05	2.24E-02
Wnt/beta-catenin Signaling	6.03E-05	1.58E-08
Hypoxia Signaling in the Cardiovascular System	6.61E-05	3.55E-02
T Cell Receptor Signaling	8.51E-05	2.69E-02
p38 MAPK Signaling	9.77E-05	1.20E-03
Acute Phase Response Signaling	2.34E-04	4.37E-07
Hepatic Fibrosis/Hepatic Stellate Cell Activation	3.89E-04	2.51E-15
FGF Signaling	4.57E-04	9.33E-08
EGF Signaling	7.76E-04	1.29E-04
PPAR Signaling	8.91E-04	5.37E-03
PDGF Signaling	1.00E-03	6.03E-05
PPARalpha/RXRalpha Activation	1.23E-03	4.90E-04
PI3K/AKT Signaling	1.62E-03	1.62E-08
Cell Cycle: G1/S Checkpoint Regulation	2.24E-03	2.57E-04
Hepatic Cholestasis	2.24E-03	6.92E-03
IGF-1 Signaling	2.88E-03	3.31E-05
Huntington's Disease Signaling	5.13E-03	1.45E-07
Integrin Signaling	5.13E-03	5.37E-04
Apoptosis Signaling	5.62E-03	1.26E-11
Death Receptor Signaling	6.17E-03	2.82E-09
Insulin Receptor Signaling	7.08E-03	5.25E-05
Fc Epsilon RI Signaling	7.24E-03	1.38E-09
PTEN Signaling	7.59E-03	1.86E-10
IL-10 Signaling	7.94E-03	1.74E-02
Actin Cytoskeleton Signaling	1.41E-02	9.77E-03
Synaptic Long Term Depression	1.95E-02	2.57E-02
JAK/Stat Signaling	2.24E-02	6.17E-05
NF-kB Signaling	2.24E-02	2.24E-05
GM-CSF Signaling	2.45E-02	5.01E-08
IL-2 Signaling	2.57E-02	1.41E-05
Chemokine Signaling	3.02E-02	5.37E-03
VEGF Signaling	3.02E-02	1.38E-07
Xenobiotic Metabolism Signaling	4.37E-02	1.78E-02

### Genetic network analysis of miR-145 targets in metastatic versus non-metastatic cells

Previous studies have demonstrated that over-expression of miR-145 has tumor suppressor effects in non-metastatic CRC cell lines such as SW480, HCT116 and DLD1 [21,27,32]. We hypothesized that the differential steady state level of mRNA targets between metastatic and non-metastatic cells might result in this observed dual function of miR-145. We therefore investigated the expression profiles of miR-145 target genes in the isogenically matched SW480 (non-metastatic) and SW620 (metastatic) cell lines [34]. Expression levels of 12% (55/450) of the predicted miR-145 target mRNAs were found to be significantly different between these cell lines. Gene set enrichment analysis of these 55 genes identified 4 biological pathways significantly altered in the metastatic setting (Table 7). Of note, the G1/S cell cycle checkpoint (represented by *MYC *and *CCND2*) and neuregulin pathways (represented by *MYC *and *ADAM17*) were down-regulated in the SW620 cells. The expression of these genes may therefore be related to the tumor suppressor effects observed in non-metastatic cells, while their absence in SW620 cells may cause miR-145 to act on other, yet to be determined, anti-proliferative signaling targets.

**Table 7:** miR-145 targeted pathways differentially expressed between SW480 and SW620 cell lines.

Pathway	Genes (SW620/SW480)
Axonal guidance signaling	ADAM17*, ARPC5, ADAM19, EFNA3*, PLXND1
Cell Cycle: G1/S checkpoint	MYC*, CCND2*
ERK/MAPK signaling	MYC*, ATF1, ETS2
Neureguin signaling	MYC*, ADAM17*

* Denotes genes that are down-regulated in SW620 compared to SW480

## Discussion

The expression of 37 miRNAs were found to be altered in CRC clinical specimens compared with normal colon and 11 of these miRNAs showed the same pattern of expression in CRC cell line models. This indicated that CRC cell lines do retain some of the miRNA expression patterns observed in primary tumors and can act as suitable models for functional analyses of specific miRNAs. Our observation that miR-143 and miR-145 were down-regulated in CRC confirmed earlier reports [21-25]. However, surprisingly these were not found in a recent study that included a large cohort of CRC clinical specimens [26]. Such discrepancies may be explained by differences in the miRNA-detection platforms used or clinical sample characteristics. We also verified the previously reported down-regulation of miR-30a-3p [22], miR-10b, miR-30c, miR-125a, miR-1, miR-133a, and miR-195 [35].

MiR-31, miR-21 and members of the miR-17-92 cluster, and its paralogues, were shown to be up-regulated in CRC. The increase in miR-31 was reported previously [22], but was not found in several other miRNA expression studies in CRC [23,26,27,35,36]. A total of 22 miRNAs were deregulated in the early stages of CRC. These miRNAs such as miR-224 and miR-21 should be further examined for their utility as diagnostic biomarkers of CRC. For example, such biomarkers could be assayed for individuals at high risk of CRC in non-invasive tissues such as blood or stool. Indeed the utility of miR-21 as a diagnostic, prognostic, and a marker of therapeutic response has been confirmed in other studies [23,26,27,35,36]. The 6 miRNAs that displayed different expression levels between early (mostly stage II) and late stage (mostly stage III) samples should also be analyzed for their association with clinical outcome. For instance gene expression profiles have been used to predict prognosis of stage II CRC patients with the goal of upgrading these individuals to chemotherapy regimens in addition to the standard of care surgery [37]. Interestingly, the recent work by Schetter and colleagues did not identify any of these 6 miRNAs as potential prognostic markers since they only focused on miRNAs that were differentially expressed between normal and all CRC stages. Addressing these questions will require the examination of large sets of clinically relevant biospecimens. To this end, we also demonstrated reproducible detection of miRNA in clinically relevant FFPE CRC samples, indicating that miRNAs are stable in this poorly preserved material [38].

We found that miR-143 re-expression in SW620 cells converted the round, independent cells into aggregated cell masses, increased E-cadherin expression and decreased cell proliferation/metabolism, all consistent with a transition to a more epithelial-like cell phenotype. Akao and colleagues have also demonstrated a reduction in cell viability upon delivery of pre-miR-143 into the non-metastatic DLD1 or SW480 CRC cell lines, which is consistent with our observations [21]. However, in contrast to our findings, the previous reports demonstrated dose-dependent tumor-suppressor effects of miR-143. These conflicting data may be due to the different cell lines models used and/or the methods used for expressing miR-143. For instance, we generated stable clones while previous studies performed transient transfections with synthetic pre-miR-143. These factors may affect the mechanisms by which the targeted pathways are regulated.

In contrast to the miR-143 tumor suppressor effects, replacing miR-145 activity in the same cells led to an elongated cell phenotype, increased E-cadherin, and increased cell proliferation/metabolism (all consistent with a mesenchymal-like phenotype). The epithelial-mesenchymal transition (EMT) represents a critical component of the progression of carcinomas towards invasive and metastatic disease, as well as allowing epithelial cells to escape the constraints of the tissue architecture. In CRC it has been reported that EMT enhances the migratory ability of cells, leads to acquisition of autocrine growth factor signaling loops, increased expression of relevant integrins and mechanism to evade apoptosis [39]. Therefore miR-145 may be an activator of the transition to a more mesenchymal-like phenotype thus advancing tumor evolution, whereas miR-143 may contribute as a guardian of the epithelial-like state (both consistent with their opposing impacts on the metastatic cell model SW620).

The oncogenic effects of miR-145 were enhanced when cells were grown in serum-free medium suggesting that cell growth factors may affect the activity of miR-145 and/or modify the endogenous activity of pathways targeted by this miRNA. Indeed previous reports have shown that miR-145 leads to a tumor suppressor effect in non-metastatic CRC cell lines (DLD1, HCT116, SW480, LS174T) [21,27]. However, we have shown here that the steady state level of miR-145 targets is significantly altered between metastatic and non-metastatic cells. Moreover, the G1/S cell cycle checkpoint (represented by *MYC *and *CCND2*) and neuregulin (represented by *MYC *and *ADAM17*) pathways that are targeted by miR-145, were found to be significantly under-expressed in the metastatic setting. Other deregulated miR-145 targeted pathways included axonal guidance signaling and the ERK/MAPK pathways. The presence and absence of these pathways in the non-metastatic and metastatic cells may account for the dual tumor suppressor and oncogenic effects of miR-145, respectively. Members of the miR-17-92 cluster have also been shown to have either oncogenic or tumor suppressor activity depending on the cellular context in which they are expressed [40]. We have now established that miR-145 can also act in this dual manner. It should be noted however, that our results were limited to one metastatic CRC model. To further validate our findings additional metastatic models should be investigated for a similar phenotypic impact. Nevertheless, investigators contemplating the use of miR-145 as a therapeutic strategy for CRC should keep in mind that specific miRNAs may have opposing biological functions at different stages of tumor development.

## Conclusion

In summary we have screened a large set of clinical CRC samples and matched normal colorectal tissue for miRNA expression. Thirty seven miRNAs were found to be differentially expressed between normal, early, or late stage CRC and the deregulation of 11 of these miRNAs was confirmed in a panel of CRC cell line models. Larger studies will need to be performed to test the utility of these miRNAs as diagnostic and prognostic markers in CRC. We elucidated the opposing phenotypic effects of miR-143 and miR-145 in metastatic CRC cells. Bioinformatic analysis provided insight into the biological pathways controlled by miR-143 and miR-145 and comparison of gene expression profiles has indicated that miR-145 targets are differentially expressed between metastatic and non-metastatic isogenic cell line models. Pathway analysis of these differentially expressed genes identified the G1/S cell cycle checkpoint and neuregulin pathways as being significantly down-regulated in SW620 compared to SW480 cells thus providing an explanation as to why miR-145 has oncogenic effects in the metastatic context in contrast to tumor suppressor effects in the non-metastatic setting. Importantly, our results highlight that delivery of miR-143, in isolation, may be a potential therapeutic modality for CRC, and that a strategy involving over-expression of miR-145 alone should be approached with caution.

## List of abbreviations

CRC: colorectal cancer; DMEM: Dulbecco's Modified Eagle Medium; FFPE: formalin fixed paraffin embedded; IMDM: Iscove's Modified Dulbecco's Medium; MAMI: META miR:target Inference; miRNA: microRNA; nt: nucleotide; pre-miRNA: precursor microRNA; QPCR: quantitative RT-PCR.

## Competing interests

The authors are employed by companies that are in the business of commercializing diagnostics (Veridex: LD) and therapeutics (Johnson & Johnson Research: GMA, LMC, AL, RD, ME, CZ, NT; Centocor R&D: HF, MR; Johnson & Johnson PRD: KR, AB) for cancer management.

## Authors' contributions

GA and MR conceived of the study, participated in its design and coordination, and drafted the manuscript. LD, LMC, AL, RD, ME, CZ, NT, and KR performed the molecular genetic studies. AB performed the miRNA profiling experiments. HF performed statistical analyses. All authors read and approved the final manuscript.

## Pre-publication history

The pre-publication history for this paper can be accessed here:

http://www.biomedcentral.com/1471-2407/9/374/prepub

## Supplementary Material

Additional file 1 **Clinical Sample Characteristics**. Characteristics of CRC clinical samples used in the study. Click here for file

Additional file 2 **Taqman miRNA Assays**. List of 169 ABI early access miRNA Taqman assays. Click here for file

Additional file 3 **Starfire Probes**. Sequences of Starfire probes used for Northern analysis. Click here for file
